# David vs. Goliath: comparing conventional machine learning and a large language model for assessing students' concept use in a physics problem

**DOI:** 10.3389/frai.2024.1408817

**Published:** 2024-09-18

**Authors:** Fabian Kieser, Paul Tschisgale, Sophia Rauh, Xiaoyu Bai, Holger Maus, Stefan Petersen, Manfred Stede, Knut Neumann, Peter Wulff

**Affiliations:** ^1^Physics and Physics Education Research, Heidelberg University of Education, Heidelberg, Germany; ^2^Department of Physics Education, Leibniz Institute for Science and Mathematics Education, Kiel, Germany; ^3^Applied Computational Linguistics, University of Potsdam, Potsdam, Germany

**Keywords:** large language models, machine learning, natural language processing, problem solving, explainable AI

## Abstract

Large language models have been shown to excel in many different tasks across disciplines and research sites. They provide novel opportunities to enhance educational research and instruction in different ways such as assessment. However, these methods have also been shown to have fundamental limitations. These relate, among others, to hallucinating knowledge, explainability of model decisions, and resource expenditure. As such, more conventional machine learning algorithms might be more convenient for specific research problems because they allow researchers more control over their research. Yet, the circumstances in which either conventional machine learning or large language models are preferable choices are not well understood. This study seeks to answer the question to what extent either conventional machine learning algorithms or a recently advanced large language model performs better in assessing students' concept use in a physics problem-solving task. We found that conventional machine learning algorithms in combination outperformed the large language model. Model decisions were then analyzed via closer examination of the models' classifications. We conclude that in specific contexts, conventional machine learning can supplement large language models, especially when labeled data is available.

## 1 Introduction

The introduction of ChatGPT, a conversational artificial intelligence (AI)-based bot, to the public in November 2022 directed attention to large language models (LLMs). As of 2023, ChatGPT is based on a LLM called Generative Pre-trained Transformer (GPT; versions 3.5, 4, 4V, or 4o) and has proven to perform surprisingly well on a wide range of different tasks in various disciplines—including medicine, law, economics, mathematics, chemistry, and physics (Hallal et al., 2023; West, 2023; Surameery and Shakor, 2023; Sinha et al., 2023). A number of tasks in education (research) can be tackled using LLMs in general, or ChatGPT more specifically. For example, LLMs were found to be able to write quality essays in physics (Yeadon et al., 2023), simulate student preconceptions for physics concepts (Kieser et al., 2023), write reflections in nursing education contexts (Li et al., 2023), and even generate feedback that was considered equally correct and more helpful by the students compared to human expert feedback (Wan and Chen, 2024). In particular, prompt engineering with LLMs (i.e., specifically designing the inputs to the LLM) was found to notably improve the capabilities and quality of outputs, to even become so-called “zero-shot reasoners” (Wan and Chen, 2024; Kojima et al., 2022).

The use of LLMs in education (research) is, however, not without challenges. When confronted with conceptual questions, LLMs may hallucinate knowledge (i.e., present false information as facts) (Huang et al., 2023), which is then concealed by its fluent language and verbose writing style (Gregorcic and Pendrill, 2023). This issue is exacerbated by the intransparency of the decisions made by LLMs (Chen et al., 2023; Manning, 2022). Intransparency in the decision-making process of an LLM may prevent researchers from understanding the logic behind a prediction, and thus hinder them from justifying their choices for certain LLMs. LLMs also exhibit human-like biases through imbalanced training data; and the extent to which LLMs truly extrapolate beyond their training data or merely mimic patterns—in the sense of “stochastic parrots” (Bender et al., 2021; Caliskan et al., 2017; Lake and Baroni, 2023)—remains an open question. Many examples demonstrate that LLMs such as GPT-4 cannot sufficiently abstract and reason (Mitchell et al., 2023). Finally, the extensive use of LLMs significantly contributes to environmental concerns, particularly in terms of CO_2_ emissions and expenditure of energy, both by training the foundation models and with every single request passed through the model (de Vries, 2023; Dodge et al., 2022).

Consequently, we argue that the circumstances where machine learning (ML) and LLMs excel respectively should be critically evaluated to derive some guidance for researchers and practitioners. Conventional AI approaches (i.e., ML algorithms) are less complex and their decisions can commonly be explained using established procedures (Lundberg et al., 2019). Given their reduced complexity, conventional ML algorithms can be operated in a controlled manner and might not generate unanticipated outputs. For example, a trained binary classifier can by design only output two categories, whereas generative LLMs used in a binary classification problem might output the categories, however, it might also produce further textual output. Whether conventional ML or LLMs are used for solving a (research) problem in part depends on the complexity of the problem. In some contexts, e.g., fourth-grade mathematics, it was found that conventional ML can outperform LLMs on identifying incoherent student answers (Urrutia and Araya, 2023). However, this research considered the LLM GPT-3, which is now surpassed by GPT-4. Given the specific potentials and limitations with either conventional ML and LLMs it remains an open question what approach should be utilized under which circumstances.

Given the successes of conventional ML such as explanability of model decisions as well as the limitations such as the ability to tackle complex problems, and the recent advances of LLMs with their “emergent abilities” (Wei et al., 2022) and zero-shot reasoning capabilities, this study compares the performances of conventional ML algorithms and a recent LLM on a physics-specific assessment problem. Our goal is to refine our understanding of the circumstances under which either conventional ML algorithms or LLMs might be better suited solutions.

## 2 Theoretical background

### 2.1 Natural language processing with conventional ML and LLMs

Language data such as students' written responses, interview transcripts, or research articles is omnipresent in educational research, and therefore integral for theory development. Educational research often draws on content analysis as an analytical method to analyze language data. One major task in content analysis is to develop categories for certain events occurring in the language data to be analyzed, such as a student using a certain concept in an interview (transcript). The actual assignment of codes to the content is guided by a coding manual that specifies the rules for when a category applies or not (Mayring, 2000; Krüger et al., 2014). Content analysis, in particular the process of developing and assigning codes, is often very time-consuming, thus limiting the amount of content (e.g., interview data) that can be analyzed. This leads to methodological constraints. For any given language, there is a set of words that frequently appears in texts, yet a much larger number of words occurs only rarely (Newman, 2005; Wulff, 2023). Hence, rare occurrences also appear only in large text corpora, making it generally insufficient to analyze only small samples to validly identify underlying patterns in textual data. Similarly, the decisions and subjective judgments of researchers involved in the analysis process can pose challenges in validating and reproducing the results of qualitative analyses (Biernacki, 2014).

Natural Language Processing (NLP) enables the use of new statistical approaches (often based on ML) to systematically analyze large data sets that are no longer analyzable by humans alone. A powerful tool that was developed by NLP researchers are word and sentence vectors, also referred to as embeddings, which can then be further processed, e.g., by ML algorithms. In the simplest case, one can use so-called “bag-of-words” models that list all words in a document and their frequencies of occurrence while omitting positional information (Zhang et al., 2010). Limitations of 'bag-of-words' models include a missing measure of similarity between individual words as these models do not consider the particular meaning of words, and they do not consider word order. To address these limitations, artificial neural networks were trained with the aim to transform textual input into (static) embeddings, i.e., numerical vectors of generally high dimensionality, that incorporate contextual information of individual words or sentences (Mikolov et al., 2013). These embedding vectors can then be used as input features for ML algorithms in further downstream tasks. ML refers to the inductive learning of patterns from data (Rauf, 2021). Various ML techniques, such as clustering or classification, can be applied based on these embedding vectors. In early NLP research, oftentimes conventional ML approaches such as logistic regressions or decision trees were utilized to build these classifiers (Jurafsky and Martin, 2014; Manning, 2022). Despite the simplicity of these models, particularly with regard to clustering, good results can also be achieved in difficult tasks such as argumentation mining (Stede and Schneider, 2019) or classification of elements of problem-solving approaches (Tschisgale et al., 2023a).

Recently, significant advancements in the field of NLP have occurred through the training of LLMs. In contrast to simple “bag-of-words” models that merely capture word frequencies in documents, and static embedding vectors, LLMs are able to more dynamically encode and also generate language. LLMs can process textual data at a much deeper level by quantifying relationships between words (often based on co-occurrence in large training corpora). The foundation for these advancements lies in a specific artificial neural network architecture called transformers (Devlin et al., 2018; Vaswani et al., 2017) that are trained on extensive textual data. Transformers brought along a vast variety of different models (Amatriain et al., 2023), such as Bi-direction Encoder Representations for Transformers (BERT) or Generative Pre-trained Transformers (GPT). The training of transformer LLMs typically involves prediction of randomly omitted words from a given sequence of context words. Surprisingly, this relatively simple training objective enabled transformers to perform well on new tasks that were not included within the training phase especially if the LLM is also given some examples (few-shot learning) (Brown et al., 2020). Two paradigms of application are differentiated: (i) fine-tuning, i.e., the LLM is trained with labeled data to perform a task, and (ii) prompting, i.e., huge-size language models (also called foundation models) are given a few examples with blanks for the model to fill in (few-shot or zero-shot learning) (Zhao et al., 2023).

Among the most widely used, popular, and performative transformer models is the Generative Pre-trained Transformers (GPT) family developed by OpenAI (Achiam et al., 2023). As a generative transformer model, GPT relies on continuing an input string, a so-called prompt. Manipulating this prompt to achieve desirable outputs is termed prompt engineering (i.e., adding specific information to an input to influence the output) and prompt chaining (i.e., concatenating subsequent prompts and outputs to align the new outputs with the flow of conversation and incorporating prior information), and was found to enable researchers to utilize GPT models specifically for their research purposes (Liu et al., 2021; White et al., 2023). One well-known application (an assistant model, Zhao et al., 2023) of GPT models is ChatGPT, a chatbot based on the GPT-3.5 (and later the GPT-4, and GPT-4V with vision capabilities) architecture (Bubeck et al., 2023). ChatGPT was particularly trained with human feedback and prompt-response pairs to enable conversational turns. It has been shown that this fine-tuning improves the performance of LLMs in various tasks (Wei et al., 2021). ChatGPT has also made an impact in the field of education (Kasneci et al., 2023), particularly in the field of physics education (Kortemeyer, 2023; West, 2023).

### 2.2 ChatGPT in physics education

A growing number of studies in physics education explored the potential of ChatGPT to solve physics problems. Some of these studies suggested that ChatGPT is unreliable in terms of the accuracy of its answers and that inconsistencies also occur within its reasoning chains (Gregorcic and Pendrill, 2023; dos Santos, 2023). However, it is argued that this apparent weakness of ChatGPT in answering physics questions can be utilized as a learning experience to promote critical thinking skills among students (Bitzenbauer, 2023). Other studies have tested the ability of ChatGPT (varying between GPT-3.5 and GPT-4) to solve multiple-choice physics questions. One of these studies found that ChatGPT was able to correctly answer 22 out of 23 questions from the well-known “Force-Concept-Inventory” (West, 2023). Kieser et al. even found that GPT-4 is capable of mimicking various student preconceptions known from physics education research when prompted to answer the “Force-Concept-Inventory”. This opens up new possibilities for the application of ChatGPT, including augmenting data sets by adding simulated (i.e., synthetic) student responses (Kieser et al., 2023). Another possibility was examined by Küchemann et al. (2023) in a randomized controlled study comparing the characteristics and quality of physics tasks created by prospective physics teachers who used either ChatGPT or a textbook as a tool. Küchemann et al. (2023) found that students in both groups faced challenges in providing all the information necessary for solving the tasks. Moreover, the authors noted that prospective physics teachers used the tasks as provided by ChatGPT without modification in 76% of cases (Küchemann et al., 2023). Krupp et al. (2023) identified various strategies for utilizing ChatGPT as an aid in solving physics problems and obtained a result similar to that of Küchemann et al. (2023). More specifically, they found that students often employed copy-and-paste techniques and accepted the solutions presented by ChatGPT without critical reflection (Krupp et al., 2023).

Wan and Chen (2024) conducted a study on the use of ChatGPT (based on GPT-3.5) to provide feedback on students' written responses to conceptual physics questions. They utilized prompt engineering and few-shot learning techniques. Their findings indicate that ChatGPT can serve as an effective tool for generating feedback based on students' responses. Even with a relatively small number of examples in training, it is possible to use LLMs through specific prompting to significantly reduce the instructor's effort required for evaluating student responses (Wan and Chen, 2024). However, LLMs may not always be the best choice for computer-assisted assessment of student responses. Urrutia and Araya (2023) found that conventional ML algorithms were more effective than LLMs when examining text-based responses from fourth-grade students to mathematics tasks. Moreover, LLMs have been critiqued for taxing the environment in unprecedented ways regarding average energy expenditure (de Vries, 2023). Also, it is difficult to explain LLMs' decisions, e.g., the generated text of a generative LLM such as GPT. Given the size of a LLM's training corpus, the size of the LLM itself (i.e., its number of its hyperparameters), and the complexity of the training process, researchers have not come up with simple ways of inspecting and explaining the generated outputs. In contrast, conventional ML algorithms such as decision trees are much easier to explain and hence control (Lundberg et al., 2019).

In sum, LLMs are quite capable tools that can be used for many applications. However, they do not appear to be silver bullets, given their tendency to hallucinate, i.e., to present false information [ranging from 3 to 29 percent of the time, even in innocuous tasks such as textual summarization (Hughes, 2023)], and their intransparency. Conventional ML might sometimes be more advantageous. However, this is unclear for rather complex tasks, e.g., those related to physics problem solving where learners have to utilize physics concepts to solve intricate problems.

### 2.3 Physics problem solving

Physics-specific problem-solving abilities are essential for students who intend to study physics and later plan to engage in a physics-related career (Armour-Garb, 2017; Mulvey and Pold, 2020; Jang, 2015). However, students' problem-solving abilities were found to be rather poorly developed, even those of students interested in science (Docktor et al., 2015; Kim and Pak, 2002). To improve students' problem-solving abilities, explicit instruction that reflects problem-solving processes proved effective (Huffman, 1997; Gaigher et al., 2007; Mason and Singh, 2010). There exist a variety of problem-solving process models (e.g., Polya, 1945; Friege, 2001, however, they all share similar phases, among them the phase of *problem representation*. Representing a given problem from a physics perspective involves identifying relevant physics concepts as well as making simplifying assumptions and idealizations. Having constructed an adequate and convenient problem representation comprises among the most important phases in physics problem solving as it determines the solution approach. Hence, the problem representation is often regarded as the crucial phase in problem solving (Savelsbergh et al., 1997; Fortus, 2008). In science domains, however, students often lack a thorough understanding of central concepts which is necessary for a useful problem representation in particular and for successful problem solving in general (Kim and Pak, 2002; Docktor et al., 2015; Hsu et al., 2004; Leonard et al., 1996). A potential reason for this might be that school instruction more often focuses on mathematical routines instead of conceptual understanding (Mulhall and Gunstone, 2012; Gerace and Beatty, 2005).

Students with less developed problem-solving abilities profit from short guidance during the problem-representation phase that helps making the problem representation more coherent and consistent (Savelsbergh et al., 1997). In order to do so, the current state of students' problem representations needs to be assessed. Considering a typical school class consisting of about thirty students and one teacher, or a decentralized learning setting (e.g., online), providing timely feedback on each student's problem representation turns out to be an impossible task for the teacher. However, if these problem representations are available in textual form, NLP and ML methods can be used to automatically assess students' problem representations and provide adaptive feedback in the form of short prompts to improve them. In general, such computer-based feedback was shown to be effective for students' learning in various settings (Graesser et al., 2018; VanLehn, 2011; Bernius et al., 2022).

Timely assessment of a large number of problem representations in textual form is daunting for teachers. Generally, students' problem representations can be regarded as well-structured in the sense that there is a limited number of particular physics concepts that ought to be included in order to make sense of a physics situation. However, describing such physics concepts in natural language may be difficult for students since language can be ambiguous, particularly the technical language of physics. For example, students could use their everyday language to circumscribe a correct physics concept (Yore and Treagust, 2006), however, students' language use could impede identifying whether the concept was used correctly or used at all.

### 2.4 The present study

Even though LLMs were found to be valuable tools, for example within physics education (West, 2023; Kieser et al., 2023), they did not excel in all tasks, particularly those that require refined conceptual knowledge or abstraction and reasoning (Gregorcic and Pendrill, 2023; dos Santos, 2023; Urrutia and Araya, 2023; Mitchell et al., 2023). Therefore, employing LLMs may not always be the best choice for computer-based assessment of students' responses and feedback provision. In particular, assessing problem representations in textual form as outlined above might be more suitable for conventional ML algorithms or LLMs such as GPT. Conventional ML algorithms may be better suited due to increased transparency of their decision-making processes, i.e., there is an overall better explainability of the generated outputs in comparison to the more black-box behavior of LLMs. Thus, we argue that in addition to investigating the potential of an LLM for assessment purposes, it is equally important to investigate the advantages of conventional ML algorithms in comparison. Particularly when aiming to assist students in the problem-representation phase during physics problem solving, it remains unclear how LLMs and conventional ML approaches perform when trying to assess students' usage of physics concepts.

Thus, this study aimed to answer the following research questions (RQs):

RQ1: To what extent can conventional ML algorithms correctly assess students' usage of physics concepts within a physics problem-solving task in comparison to ChatGPT based on an engineered prompt and a baseline classifier?
RQ2: To what extent are decisions underlying the assessment of both conventional ML algorithms and ChatGPT explainable?

## 3 Methods

### 3.1 Study context

This study is based on data from the WinnerS research project, which analyzed major problem-centered science competitions in Germany, including the German Physics Olympiad (Petersen and Wulff, 2017) in which physics problem solving plays a major role (Tschisgale et al., 2024). In addition to collecting data of Physics Olympiad participants, the research project also gathered data of non-participating students that were comparable to participating students in terms of age and school type. In total, there were 444 student responses to a problem-solving task detailed below. On average, a response contained approximately 266 characters. The complete data set which includes all student responses (Physics Olympiad participants and non-participants) is freely accessible in an Open Science Framework (OSF) repository (Tschisgale et al., 2023b).

### 3.2 Problem-solving task

**Figure 1:**
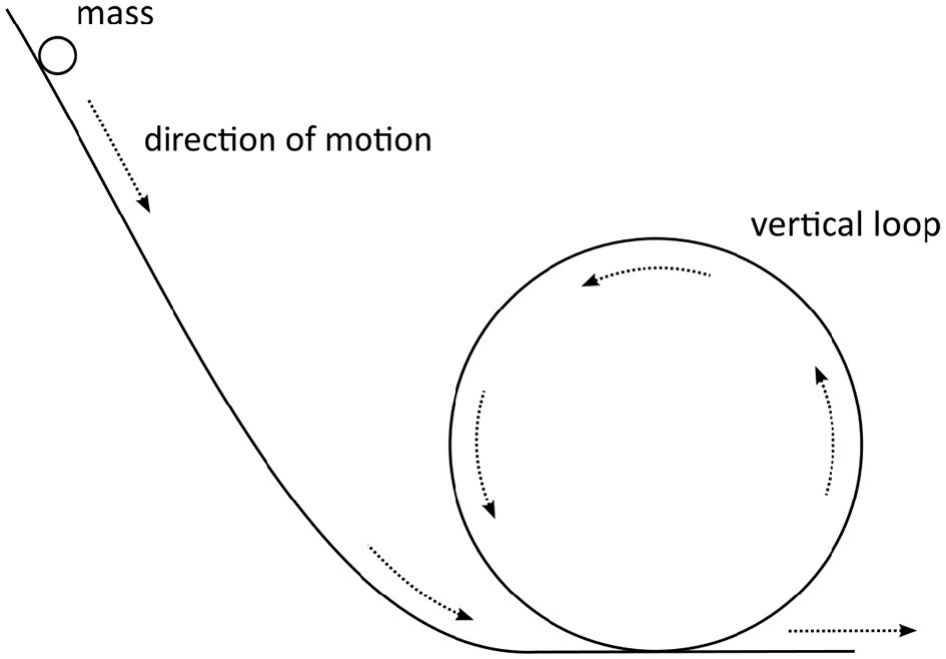
Illustration of the vertical loop as presented in the online assessment.

The task's instruction was as follows (translated to English by the authors): A very small mass slides along a track with a vertical loop (see Figure 1). The mass starts from a height above the highest point of the loop. Assume the motion to be frictionless. Determine the minimum starting height above the lowest point of the loop necessary for the mass to run through the loop without falling down. Describe clearly and in full sentences how you would solve this problem and what physics ideas you would use.

Instead of letting students solve the physics problem-solving task the typical (mathematics-centered) way, they were instructed to write full sentences and particularly focus on the relevant physics ideas. By prompting students to write full sentences, we intended to reduce the amount of mathematical representations used by students as students' physics problem solving typically involves using formulas and equations as representatives or clarifications for specific physics concepts. By saying to focus on physics ideas, students should mainly remain within the phase of problem representation (Friege, 2001), i.e., students' textual descriptions should primarily entail physics assumptions and idealizations as well as explanations around physics concepts that are regarded important for the task. An ideal student response may therefore entail simplifying assumptions such as considering a point mass, neglecting friction, and modeling the loop as circular. Under these assumptions, solving the loop task involves exactly two physics concepts: (1) the law of conservation of energy and (2) centripetal forces as the cause of circular motions (or considering an equilibrium of the centrifugal and gravitational force in a co-moving reference frame). The crux of this specific problem-solving task is to apply these concepts to the uppermost point within the loop. If the mass is just able to pass the loop, the gravitational force on the mass acts completely as the centripetal force at the uppermost point. This idea in combination with the law of conservation of mechanical energy, i.e., that the initial potential energy of the mass due to its starting height equals the potential and kinetic energy at the loop's uppermost point (neglecting rotational energy, given that only frictionless sliding is considered), in theory allows to solve this task using basic mathematics.

### 3.3 Coding manual

Generally, two fundamental physics concepts are necessary for solving the introduced physics problem-solving task: the law of conservation of energy and the concept of centripetal forces. Two coders searched for these two physics concepts within each student's textual response and marked the corresponding segments. It should be noted that a segment's start and ending did not need to align with the start and ending of a sentence. Therefore, segments could correspond to few words within a sentence or even go beyond multiple sentences. Due to segments' free start and ending, determining a measure of interrater reliability proves difficult. A proposed reliability measure that mitigates this issue is the gamma agreement (Mathet et al., 2015).

Here is an example of the coded segments in a student response. We indicated the segments containing the energy or force concept using brackets and denoted the exact concept in italic font:

**Example 1:**

The approach is to first select the equilibrium of forces. [*Force concept:* The centripetal force at the uppermost point of the loop must be at least as great as the weight of the mass.] [*Energy concept:* The minimum starting height can then be calculated using the law of conservation of energy (kinetic and potential energy within in the loop) using the required potential energy at the starting point].

**Example 2:**

[*Force concept:* The centrifugal force in the loop depends on the ball's velocity, mass and the radius of the loop and must exceed the gravitational force at the loop's uppermost point.] [*Energy concept:* The ball's velocity in the loop's uppermost point depends on the height difference between the ball's starting point and the loop's uppermost point.] Plug formulas into each other, rearrange, and determine the minimum height difference with regard to the loop's uppermost point at which the total force = 0. The result is the loop's uppermost point plus the height difference.

Example 1 highlights that we only coded text segments in which the physics concepts are directly applied to the task, e.g., simply stating “conservation of energy holds” was not enough. For the force concept, it was important that students specified the relevant forces that act at the highest point of the loop. For the energy concept, it was important which forms of energy occur and how they relate. If the concepts are described in a too general manner, they are not coded (see first sentence of the first example). Example 2 illustrates that while coding the force concept, we also allowed text segments about an equilibrium of forces (involving the centrifugal force), which is only correct in the co-moving reference system of the mass. However, the data set showed that this approach was frequently used among students, which is why it was also considered as a correct usage of the force concept.

The data was coded by two independent human raters with physics expertise (one graduate student and the first author). Both raters coded a subset of the data. Afterwards, disagreements were discussed and reconciled. Finally, the entire data set was coded by both raters and gamma agreements was calculated to be .67 which we consider reliable [comparing it to thresholds for Cohen's kappa and Krippendorff's alpha (Landis and Koch, 1977)].

### 3.4 Data pre-processing

In order to simplify the ML problem to a classification problem on fixed units, we decided to split each response into its constituting sentences. Checking the output of this segmentation procedure revealed that it seemed to work well and provided an accurate segmentation of the original student responses. The original document-level human coding of the physics concepts was transferred to sentence level in the following manner: If a word in a sentence belonged to a coded segment in the original document-level response, the whole sentence was assigned as including the physics concept. For example, if in the original document-level coding a physics concept spread out over two sentences (i.e., the coded segment began in the first sentence and ended within the second sentence), both sentences would be considered as incorporating the concept on sentence-level. We manually sorted out sentences where the automated sentence splitting was incorrect or where the coding no longer made sense after splitting. This way, we ended up with 284 sentences that took into account the force coding and 288 sentences for the energy coding (see Table 1). There are 53 sentences that contain the energy concept and 40 sentences that contain the force concept (we refer to them as the positive class). We are therefore dealing with an unbalanced data set.

**Table 1:** Class distributions for energy and force codings.

	**Positive class**	**Negative class**	**Total**
Energy coding	53	235	288
Force coding	40	244	284

In summary, apart from the automatic sentence segmentation and the corresponding transfer of codings from document to sentence level, no further pre-processing, such as spelling correction or removal of formulas, was conducted. The labeled sentence corpus created this way was then used to answer our research questions.

### 3.5 Analyses procedures

#### 3.5.1 RQ1: comparing conventional ML algorithms, ChatGPT, and a baseline model

In RQ1, we aimed to assess the performance of three different approaches for correctly assessing students' usage of physics concepts within a physics problem-solving task. Each approach corresponds to a specific classifier built to predict whether a sentence of a student response either includes the energy concept or the force concept.

**Figure 2:**
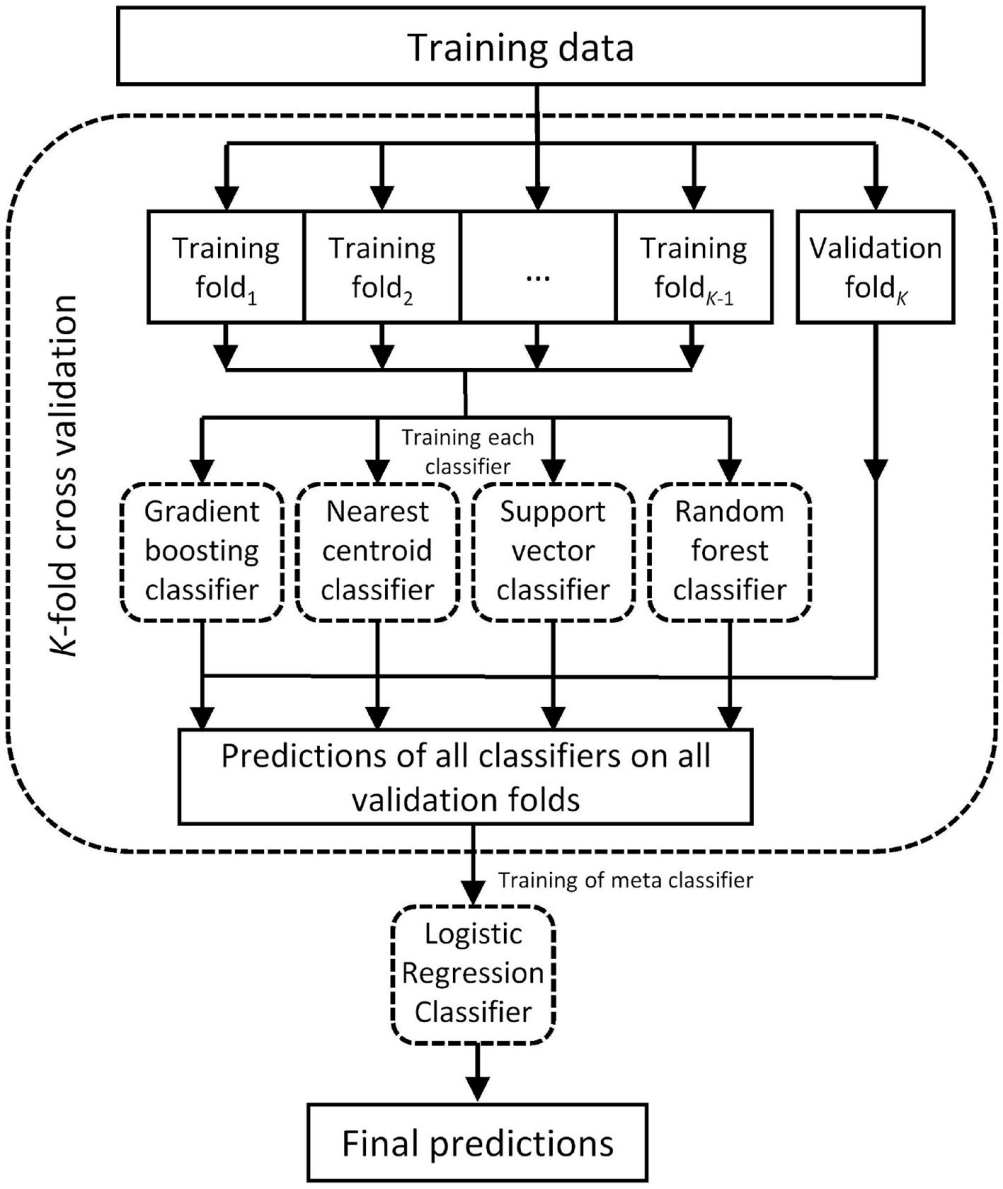
Illustration of the ML-based ensemble classifier, based on Raschka (2018).

For the conventional ML approach, we employed a stacking classifier, which is a special case of so-called ensemble classifiers (Dietterich, 2000). Such an ensemble classifier combines the predictions of multiple ML-classifiers in order to improve generalizability and robustness over an individual classifier by combining the advantages of the individual classifiers. In this study, we chose a stacking classifier from the mlxtend library (Raschka, 2018) which is written in the Python programming language (as are all other libraries that are referred to later on). The classifier inherently includes some form of cross validation (Bishop, 2006). This logic of the classifier is depicted in Figure 2. This stacking classifier consisted of four base classifiers and involved a 5-fold cross validation. Specifically, we chose a gradient boosting classifier, a nearest centroid classifier, and a support vector classifier from scikit-learn (Pedregosa et al., 2011). The fourth base classifier differed for the energy and the force concept. While a balanced random forest classifier from imbalanced-learn (Lemaître et al., 2017) was used for the energy-specific classifier, a random forest classifier from scikit-learn (Pedregosa et al., 2011) was used within the force-specific ensemble classifier. The decision for the base classifiers within the stacking classifier was based on prior experimentation on model performance.

In order to make students' textual responses in the form of sentences processable for any ML-based classifier, we generated multiple features (i.e., numeric representations of the sentences) based on the input sentences. More specifically, we used TF-IDF (term-frequency inverse-document-frequency) weighted word unigrams, character n-grams of the size 3 to 6, and sentence embeddings from the spaCy library (Honnibal and Montani, 2017) for feature generation. A sentence embedding is a numerical representation of a sentence in the form of a vector (of generally high dimensionality) that captures the meaning of a sentence. We used sentence embeddings that originate from the de-core-news-lg model. These embeddings are generated by calculating the average of the vectors of the individual tokens. TF-IDF weighting takes into account both the relative frequency of a word among all documents and the inverse frequency of the word in all documents (Qaiser and Ali, 2018). Moreover, two additional binary features were included. The first feature checked whether the sentence only contains a formula. The formulas could be identified by searching for special characters. The second feature checked whether the sentence contained words from a predefined word list. One such word list was created for each relevant physics concept (energy and force). These lists were selected according to which words frequently occur in the positive class but not in the negative class. Manual attempts were also made to identify patterns in the data. As a result, words were added to the word lists^1^. The energy-specific ensemble classifier used both additional features while the force-specific ensemble classifier only used the word list, as prior experimentation showed that the other feature (the presence of a formula) minimally decreased performance.

We also want to point out that both ensemble classifiers, which are in the following referred to as ML-based classifier (Energy) and ML-based classifier (Force), were tested using variations in pre-processing, used features, and the combination of classifiers. Pre-processing experiments included lowercasing, lemmatizing (transforming words to their base form), and the removal of punctuation, special characters, and stop words. While the force classifier achieved better results with lemmatization, the results of the energy classifier improved through the removal of stop words instead. Both performed better without punctuation and special characters. Lowercasing had no positive influence on the performance, so the upper case characters were left unchanged in the end. Other approaches included BERT sentence embeddings (Dietterich, 2000) from the transformers library (Wolf et al., 2020) which were discarded because they resulted in a high precision score, but a low recall score for the positive class. Oversampling methods like SMOTE (Chawla et al., 2002) and undersampling techniques to address the class imbalance were not expedient either.

For the ChatGPT-based approach, we used ChatGPT (gpt-3.5-turbo-instruct with standard settings) as a binary classifier by means of prompting. We used the Python programming language and ChatGPT's API to automatically store the binary outputs of the ChatGPT-based classifier (i.e., “Yes” or “No”) in a list for further processing. Specifically, we used the following prompts (translated into English by the authors):

**Energy-specific prompt:**

Can you tell me whether the following sentence from a learner contains a statement about the law of conservation of energy? Sentence: “(...)” Please answer yes or no first and do not provide any reasoning.

**Force-specific prompt:**

Can you tell me whether the following sentence from a learner contains a statement about the balance of forces?^2^ Sentence: “(..)” Please only answer yes or no first and do not provide any reasoning.

We then attempted to improve the ChatGPT-based classifiers' performance by using few-shot-learning, i.e., by providing ChatGPT sample sentences and their assigned class through the corresponding prompt. For the energy classifier, we have selected sentences that do not contain the energy approach, and for the force classifier, sentences that contain the force approach. Specifically, we used the following prompts (translated into English by the authors):

**Energy-specific few-shot-learning prompt:**

Can you tell me whether the following sentence from a student contains a statement about the law of conservation of energy? Sentence: (...) To help you, here are some examples that do not contain the law of conservation of energy: 1. At the highest point of the loop, the speed must be high enough for the radial force, which is proportional to the square of the speed and inversely proportional to the radius, to be at least equal to the weight of the ball. 2. In this case, start height = loop height, because the energy is converted immediately. 3. The mass would have to fall from a starting height that is at least as high as the highest point of the looping Please only answer yes or no and do not provide any reasoning.

**Force-specific few-shot-learning prompt:**

Can you tell me whether the following sentence from a learner contains a statement about the equilibrium of forces? Sentence: (...) "To help you, here are some examples that contain the force approach:

1. At the highest point of the loop, the speed must be high enough for the radial force, which is proportional to the square of the speed and inversely proportional to the radius, to be at least equal to the weight of the ball.

2. At the top of the loop, the radial force must just compensate for the weight of the mass so that the mass does not fall downwards.

Please only answer yes or no and do not provide any reasoning.

For the baseline approach, we established a simple rule-based classifier that assigned a sentence to the positive class (i.e., sentence includes one of the central physics concepts) if this sentence included the character string “energie” or “kraft” (German words for “energy” and “force,” respectively). We therefore refer to this rule-based classifier as word-checking classifier.

To evaluate the performance of each classifiers, metrics such as accuracy (proportion of correctly assigned sentences) can be used. However, solely focusing on classifiers' accuracy is not sufficient to evaluate performance, particularly if data sets are unbalanced as in our case. Unbalanced means that a specific class (e.g., sentence includes energy concept) occurs much more frequently or rarely than the other classes (e.g., sentence does not include energy concept). In such cases, further performance metrics that also take into account the type of incorrect classification (i.e., false-positive or false-negative) are needed. Therefore, we also computed precision, recall, and F1 values as further performance metrics. Precision measures the accuracy of the positive predictions made by a classifier. In our case, precision answers the question: “Of all sentences that were predicted to include the energy (force) concept, how many sentences actually include the energy (force concept)?” Recall (or sensitivity) measures the completeness of positive predictors. In simpler terms and framed to our context, recall answers the question: “Of all sentences that actually include the energy (force) concept, how many did the classifier correctly identify?” The F1 score is the harmonic mean of precision and recall, providing a single metric that balances the trade-off between both precision and recall. All these metrics range from zero to one and a higher value generally indicates better classification.

#### 3.5.2 RQ2: making model decisions explainable

An essential aspect that builds trust in AI models and opportunities for researchers to improve models is the possibility to understand why the model makes certain decisions (Zhao et al., 2023). This is also known as “explainable AI” (Lipton, 2018). Explainability refers to the ability to “explain or present the behavior of models in human-understandable terms” (Zhao et al., 2023, p. 1). There are many different ways to illuminate different aspects of explainability for LLMs in the fine-tuning paradigm such as calculating the attribution scores for each input that indicate the respective impact on the classification (Zhao et al., 2023). For LLMs in the prompting paradigm, there also exist some methods which are necessarily constrained if models are closed-source such as ChatGPT (Zhao et al., 2023). Besides access restrictions, with such LLMs as GPT-4 it is not yet possible to entirely explain internal workings of the models and the generated outputs in human-understandable terms. This is because these language models are trained on extensive data and due to their complexity and diversity of language patterns, they can produce unpredictable results. Even if there are approaches to making the transformer architectures on which large language models are based transparent (Vig, 2019). It is unclear whether these methods have an impact on trust in AI decisions (Conijn et al., 2023). There are no explicit rules or methods to predict the exact output in advance. Instead, assessing the quality of the output relies on experience and the model's past behavior, based on previous results or benchmarks. In short, the versatility and complexity of LLMs makes it difficult to determine the exact output in advance, and one must rely on experience to evaluate their performance.

One method of making model decisions more explainable is through analyzing model outputs. In such cases one distinguishes local explanations and global explanations (Schrouff et al., 2021; Zhao et al., 2023). Local explanations address the question of why a specific student response is categorized in a particular way, while global explanations try to answer the question of why a whole group of student responses is categorized in a particular manner, i.e., one tries to understand the model in more general terms. In this study, we chose a global approach because we were interested in overall model decisions which might provide insights into students' text composition processes (e.g., which words are particularly predictive for a certain classification). First, we grouped individual sentences into separate documents based on their classifications into a specific category (e.g., false positive). Thus, we obtained four separate documents. Then, to identify patterns which might explain the models' classifications, we computed a term-frequency inverse-document-frequency (TF-IDF) score for every word in each of the four documents. Words with the highest TF-IDF scores in each category-specific document can then be considered as characteristic for this specific category (e.g., false positive), which is why we refer to them as category-specific keywords. Finally, these category-specific keywords may reveal patterns that provide an understanding of the models' decision-making. Analyzing the words that are assigned to a specific class therefore provides an approach for interpreting the assignment, as the words have a strong influence on the classification. As both the machine learning approach and the large language model approach are based on the embeddings of the tokens that make up the words. The words therefore have a major influence on the model output.

## 4 Results

### 4.1 RQ1: comparing correctness of conventional ML and ChatGPT

In Tables 2, 3 we summarized the classification performance for the ML-based classifier, the ChatGPT-based classifier, and the Baseline classifier. Through the combination of the different ML classifiers (ensemble classifier) and fine-tuning for the conventional ML algorithms, a final F1 performance of 0.74, and 0.82 for energy and force, respectively, could be achieved. Precision and recall were always above 0.69 for both ML-based classifiers.

**Table 2:** Performance metrics of the classifiers for the energy concept.

**Classifier**	**Accuracy**	**Precision**	**Recall**	**F1**
Word-checking	0.83	0.55	0.81	0.66
ChatGPT-based classifier	0.35	0.20	0.90	0.33
ChatGPT-based classifier incl. few-shot	0.35	0.20	0.88	0.27
ML-based classifier	0.88	0.69	0.79	0.74

**Table 3:** Performance metrics of the classifiers for the force concept.

**Classifier**	**Accuracy**	**Precision**	**Recall**	**F1**
Word-checking	0.90	0.60	0.80	0.69
ChatGPT-based classifier	0.32	0.16	0.88	0.27
ChatGPT-based classifier incl. few-shot	0.28	0.17	0.88	0.27
ML-based classifier	0.94	0.91	0.75	0.82

**Figure 3:**
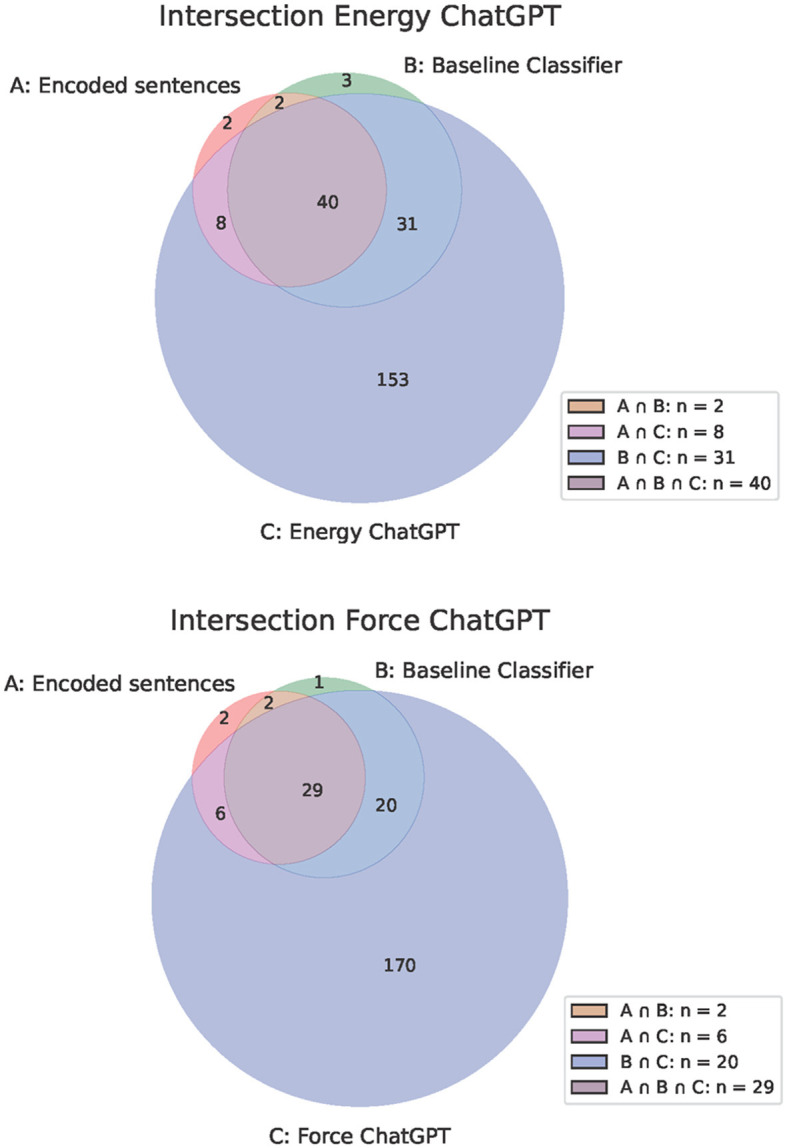
Overlapping classifications of the ChatGPT-based classifier, the baseline word-checking classifier and the encoded sentences.

In contrast, both ChatGPT-based classifiers only achieved a low precision (energy: 0.20; force: 0.16), i.e., both classifiers incorrectly assigned a large proportion of sentences that did not contain the relevant physics concepts as including the concepts. Hence, it seemed that ChatGPT tended to classify sentences as including the energy or force concept. This can also be seen by inspecting Figure 3. The Figure is used to illustrate the relationships in codings between different sets. The individual diagrams show three circles that overlap and form a total of seven different areas. Each circle represents a set, and the overlaps show the common elements between the sets. Circle A represents the set of coded sentences. Circle B represents the set of sentences that are assigned to the positive class by the baseline classifier. Circle C represents the set of sentences assigned to the positive class by the ChatGPT classifier. The overlapping areas between two circles show the elements that are contained in both sets, but not in the third set. The area in which all three circles overlap represents the elements that are contained in all three sets. The largest circle illustrates the sentences that were assigned to the positive class (i.e., energy or force is in sentence) by the ChatGPT-based classifier. The ChatCPT based classifier therefore assigns a large number of sentences to the positive class.

We also attempted to improve the ChatGPT-based classifiers' performance by means of few-shot learning, i.e., by showing sample sentences including correct class labels to ChatGPT within the prompt. For the ChatGPT-based classifier for the force concept, the F1 score remained unchanged at 0.20 (see Table 3). For the ChatGPT-based classifier for the energy concept, the F1 score actually dropped through the few-shot-learning approach from 0.33 to 0.27 (see Table 2). Both ChatGPT-based classifiers thus performed worse than the baseline word-checking classifier.

**Figure 4:**
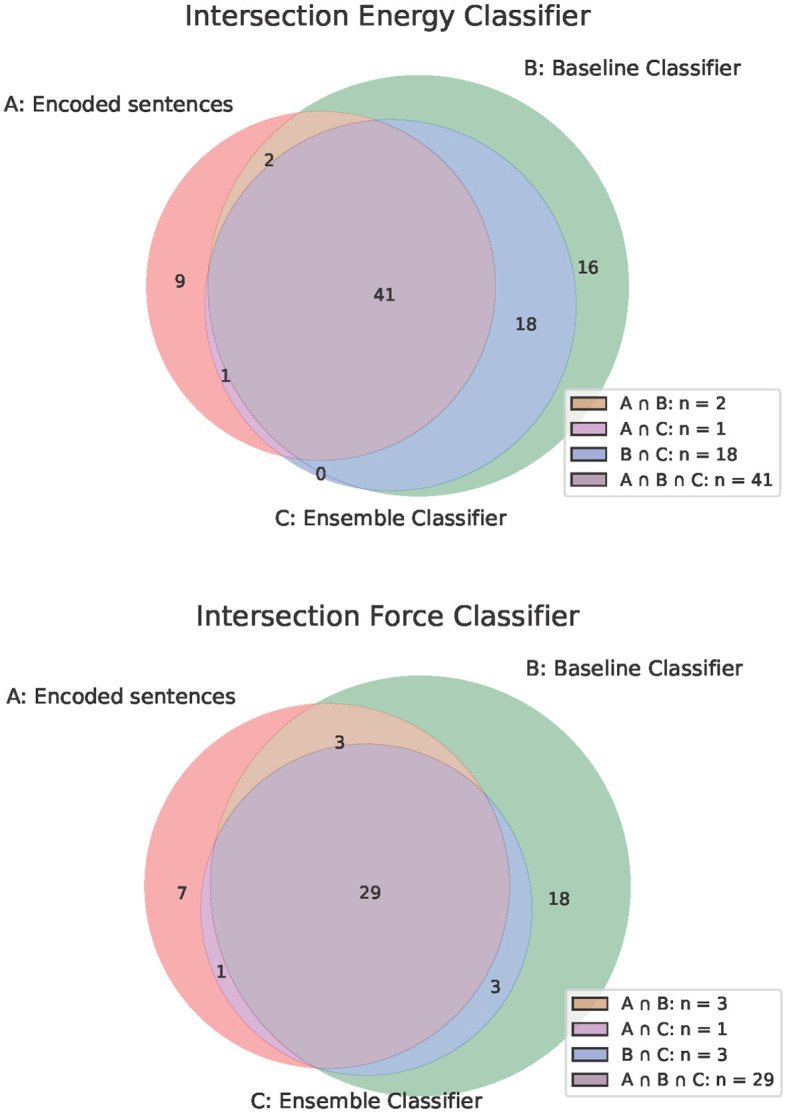
Overlapping classifications by the ML-based classifier, the word-checking classifier and the encoded sentences.

Compared to the two word-checking classifiers, which only considered whether the strings “energy” or “force” were present in a sentence, both ML-based classifiers showed satisfactory results in the F1 value (see Tables 2, 3). The low precision value of the ML-based classifier for energy, compared to the ML-based classifier for force, suggests that the model incorrectly classified some text segments as positive. This could be due to certain student responses containing words related to “energy,” but without explicitly demonstrating the application of the energy conservation principle in the context of the task. This potentially posed a challenge for the classifier. In Figure 4, the number of sentences for the various intersections between encoded sentences, the ML-based classifier, and the word-checking classifier are depicted. We can read from the figure that a total of 11 sentences that were positively coded were not recognized by the ML-based classifier for energy. A comparison of Figures 3, 4 illustrates that the ML-based classifications are much closer to the coded sentences by humans (which we considered as the gold-standard).

### 4.2 RQ2: making model decisions explainable

#### 4.2.1 ChatGPT-based classifier

In Figure 3 we see that the ChatGPT-based classifiers tended to assign sentences to the positive classes. This leads to poor precision, recall, and F1 values for these classifiers. Due to these poor performance metrics, it is not possible to interpret the outputs of the classifier more precisely.

#### 4.2.2 ML-based classifier

Now we want to evaluate the consistency of the different classifiers, i.e. the extent to which two or three different classifiers assign a sentence to the same categories or to different categories. In Section 3.5.2, we explained that by understanding previous decisions, you can gain clarity about how a classifier works. For better comparability of the classifiers, it is advisable to analyze the different assignments of a sentence by the classifiers. There are eight different ways in which a sentence can be classified:

1. The sentence can be positively or negatively coded.
2. The baseline classifier can classify it as positive or negative.
3. The ML classifier can classify it as positive or negative.

As each of these three decisions is independent, there are a total of eight different combinations. These different combinatorial possibilities result in eight disjoint sets. These sets are shown in Table 4 and the intersections are also shown in Figure 4.

**Table 4:** Number of sentences in intersections between baseline word-checking and ML-based classifiers for force and energy.

**Row no**.	**Encoded sentences**	**Word-checking classifier**	**ML-based classifier**	**# sentences (energy)**	**# sentences (force)**
1	0	0	0	201	223
2	0	0	1	0	0
3	0	1	0	16	18
4	0	1	1	18	3
5	1	0	0	9	7
6	1	0	1	1	1
7	1	1	0	2	3
8	1	1	1	41	29

**Figure 5:**
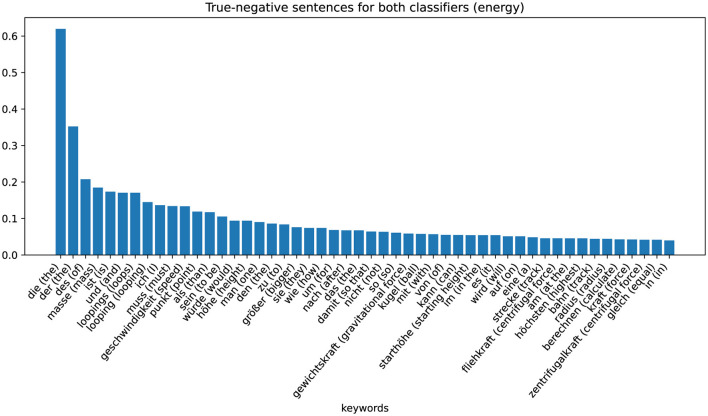
Category-specific keywords in true negative classified sentences (energy classifier).

**Figure 6:**
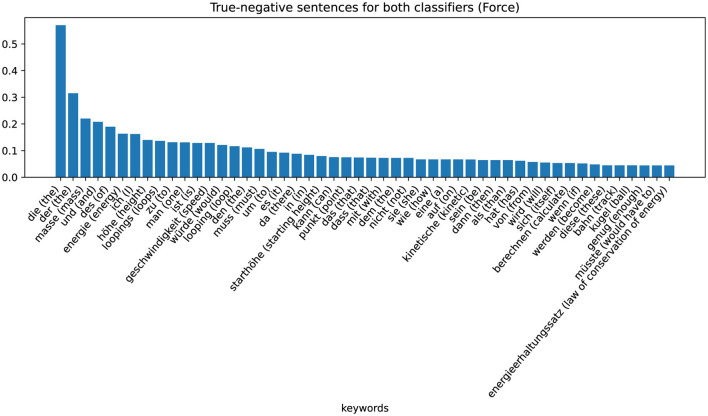
Category-specific keywords in true negative classified sentences (force classifier).

The zeros or ones in the cells of the table indicate whether the sentences are assigned to the positive (“1”) or negative ('0') class by the respective classifier (column). By analyzing the characteristics of these eight different sets, we can recognize patterns and gain information on why the classifier makes certain decisions. The largest set is that of non-coded sentences that are not assigned to the positive class by either the word-checking classifier or the ML-based classifier (row one in Table 4). These sentences are examples in which the classifier has classified correctly. The keywords extracted with TF-IDF values are shown in the Figure 5 (Energy) and Figure 6 (Force). Figure 5 shows these sentences that are correctly assigned to NOT contain the energy approach, for example, words that describe the looping or words that describe the force approach. Figure 6 shows that sentences that are correctly not assigned to the force approach contain words that can be assigned to the energy approach: “energy,” “kinetic,” “law of conservation of energy.” We can therefore interpret that the classifier assigns the sentences of the positive class of the force approach to the negative class of the energy approach and vice versa.

For both approaches, there are no cases where the ML-based classifier assigns the sentence to the positive class, while the sentence is negatively coded and does not contain the terms “energy” or “power” (see the second row in Table 4). The third row in Table 4 is an interesting case, as these sentences are examples where the ML-based classifier performs better compared to the word-checking classifier. The following examples are included in the set for the energy classifier:

Then I use the law of conservation of energy, neglecting the friction of the mass.

The mass must have enough energy at the highest point of the loop so that the centrifugal force keeps it on track.

For the Force-Classifier there are the following examples:

There is a centrifugal force, the mass should have a constantly increasing speed that increases during the loop or afterwards but is smallest at the highest point of the loop.

The centrifugal force can be calculated using the speed which results from the kinetic energy equation.

These sentences are all examples in which it is not clear that the learners are applying the conservation of energy concept and the force concept to the context of the task, but the string “energy” or “force” still appears in the sentence. The classifier has learnt to assign these sentences. However, there are instances where the ML-based classifier does not outperform the word-checking classifier. In row four of Table 4, sentences are displayed that are not encoded as positive, yet both the word-checking classifier and the ML-based classifier predict the sentence as positive. For the ML-based classifier (energy) there are the following examples:

At first one should know that the law of conservation of energy plays an important role here, then one plugs the energy into a formula and gets an equation.

The kinetic energy is proportional to the mass and the square of the velocity, the rotational energy is proportional to the mass, the square of the radius (moment of inertia) as well as the square of the angular velocity.

The answers mention words such as conservation of energy, but do not apply them to the context of the task. In the second example, physics formulae are described in words, but this does not describe a physics approach applied to the context of this task. The fifth row in Table 4 shows sentences that are difficult to identify because they are positively encoded, but the word-checking classifier assigns the sentences to the negative class. Examples are:

However, it should be noted that the mass is slowed down by gravity on the way up.

The height depends on the weight of the mass and the radius of the loop, because if one is changed, the speed and the distance change, thus the centrifugal force resulting from the starting height must be adjusted.

These types of sentences are difficult for the classifier to assign correctly, as they are very specific but still elaborate on conservation of energy. For each classifier, there exists a particular sentence in the data set where both ML-based classifiers predict a true positive outcome, while the word-checking classifier predicts a negative outcome (see sixth row of Table 4). These sentences are:

The following holds: E(kin) + E(pot).

and

Equilibrium of forces at the highest point of the loop, velocity via energy approach.

The first example captures (parts of) the conservation of energy expressed in a mathematical formula. At this point it should be noted that, based on this example, it is quite difficult to conclude that the ML-based classifier has now “learned” this mathematical expression. It could just as well be that other formulae are also positively classified, although they represent completely different physics content. The second example was not recognized by the word-checking force classifier because the character string “kraft” (force) does not appear in the sentence. Nevertheless, vocabulary similar to force was used and the ML-based classifier predicts positive. We also have sentences in the data set where the ML-based model performs worse than the baseline classifier (see sixth row of Table 4).

This in turn means that at least this amount of energy must be available at the beginning.

Otherwise the mass does not have enough energy to pass through it.

For the ML-based classifier (energy) this can be traced back to errors in the sentence split. For the ML-based classifier (force), these are rather colloquial answers that do not use physics-specific vocabulary and are probably therefore difficult for the ML-based classifier to recognize.

To prevent the mass from falling out of the loop, the force directed upwards at its highest point must be at least as great as the force directed downwards.

The mass starts as high as twice the height of the loop to exploit the centrifugal force and be pressed with enough momentum against the track of the loop.

**Figure 7:**
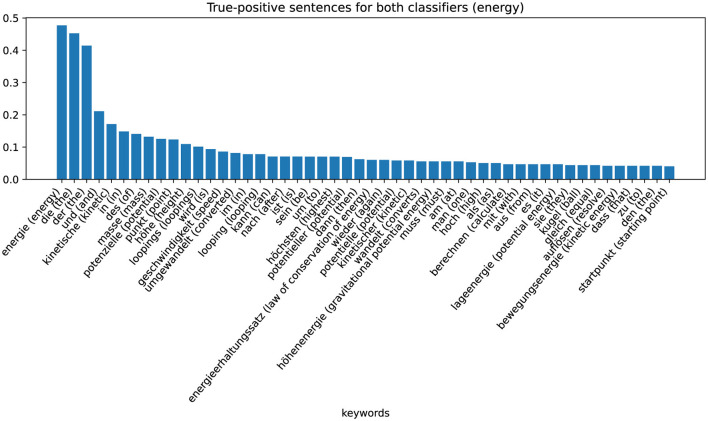
Category-specific keywords in true positive classified sentences (energy classifier).

**Figure 8:**
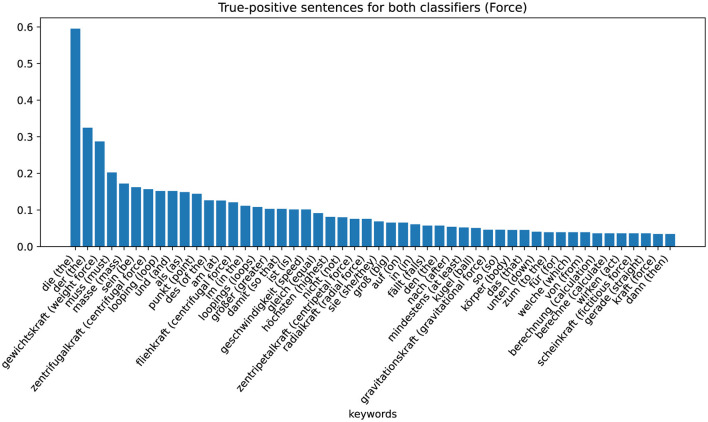
Category-specific keywords in true positive classified sentences (force classifier).

The last group is the group in which word-checking and ML-based classifiers classify correctly positive. From this data set of responses, we extract the keywords again using TF-IDF values. The results are shown in Figure 7 (Energy) and Figure 8 (Force). Figure 7 shows that for sentences that are correctly assigned to the energy approach, an important word is, in fact, “energy.” Just like the adjectives potential or kinetic. For sentences that are correctly assigned to the force approach, the most important keyword (besides the German articles “die” and “der”) is “weight force” (see Figure 8).

## 5 Discussion

In this study we sought to evaluate and compare the performance of conventional ML algorithms and an LLM-based approach to solve a well-defined binary classification problem in the domain of physics education. We found that for this specific classification task, the conventional ML algorithms outperformed the LLM classifier based on GPT 3.5 (turbo), even when simple prompt engineering techniques are employed to potentially improve GPT's performance.

This findings was somewhat unexpected. After all, LLMs were shown to perform well on a broad range of benchmark problems, and prompt engineering and prompt chaining was shown to enhance output correctness of LLMs (Chen et al., 2023; White et al., 2023). Because we do not consider our problem to be too difficult, and human interrater agreement was satisfactory, conventional ML algorithms excelled at this classification problem. It has to be said, though, that we only tried a simple prompt engineering approach, and it is quite likely that more sophisticated prompt engineering techniques would improve the classification performance in our context. Some authors suggest that when a LLM fails to perform a task, it does not necessarily indicate that the LLM is incapable of solving the task. Instead, it may simply mean that the appropriate prompt has not yet been found (Polverini and Gregorcic, 2024; Bowman, 2023). Be that as it may, this was not the main point of this study, and designing prompts might take considerable time which then would suggest the use of the conventional ML algorithms eventually. We also recognize the fundamental challenges (hallucination, explainability, resource expenditure) of LLMs and sought to estimate to what extent and under which circumstances conventional ML algorithms that are optimized in standard ways could reach similar performance. In fact, they outperform ChatGPT noticeably in our context–even the Baseline classifier. This indicates that in fact conventional ML algorithms should also be considered if researchers want to tackle specific research problems with well-specified tasks and reliably coded data. However, conventional ML algorithms are more difficult to adopt to novel contexts and LLMs such as GPT can be considered versatile tools that, beyond assessment as in our context, have a broader scope of applicability (Wan and Chen, 2024). This does also not mean that LLMs cannot be used in classification contexts. Rather, researchers would typically train foundation models in a fine-tuning paradigm to utilize LLMs for classification problems (Devlin et al., 2018).

The ecological footprint of LLMs remains an issue, where conventional ML algorithms as of now are much more resource friendly. Moreover, LLMs tend to perform better in English (Etxaniz et al., 2023). Since not all researchers might have the capacities to train LLMs for specific languages from scratch, conventional ML algorithms might present a valuable option to achieve good performance in non-English tasks.

### 5.1 Limitations

Even though conventional ML algorithms are more resource friendly this does not necessarily mean that they are more useful. One rarely needs to only assess students' concept use in one specific problem-solving task. This also relates to a limitation of our study. We only investigated students' responses to one particular physics problem. While concept use of energy and force is useful throughout physics, however, we cannot rule out that our classifier only performs well for this specific task. Yet, the programming code could be re-used for training a similar classifier for another problem, if a coding manual and coded data is available. This limits the scalability of the conventional ML approach.

Another limitation relates to the generalizability of our findings to other student populations. The investigated student population is not representative of a broad student population. Almost all Physics Olympiad participants and all non-participants attended academic track (*Gymnasium*) and were from higher grade levels. It remains unclear how the investigated models would have performed on responses of less performant students as their responses might have involved for example more colloquial wordings, student preconceptions, and spelling mistakes. All these aspects might have an influence on the performance of LLM and ML algorithms.

Other limitations relate to our data pre-processing and application of the algorithms. We only trained and validated the conventional ML algorithms at sentence level to form a well-posed classification problem. However, student answers should be considered holistically, because the meaning can only be understood across several sentences. Moreover, we cannot rule out that other conventional ML algorithms might have exhibited better performance or that further modifications of the prompts to the LLM would enhance classification performance (Wan and Chen, 2024). Future research should apply prompting strategies that have been found to be performant for such contexts. Yet, these strategies also require substantive domain knowledge, and hence they are no silver bullet that automatically solve classification problems.

Finally, there are many different strategies to also inspect decisions of LLMs (Zhao et al., 2023). For example, in the fine-tuning paradigm attributions for the input features could be calculated that then indicate how much a certain input feature contributed to an output. However, these approaches require a large amount of technical sophistication and are much better worked out for LLMs in the fine-tuning paradigm as compared to generative LLMs with prompting. Here, prompting would also require substantive domain knowledge to investigate and understand model outputs.

## 6 Conclusions and implications

LLMs are sometimes referred to as zero-shot reasoners (Kojima et al., 2022) and can perform a variety of tasks. They have the ability to generalize, meaning that they can solve tasks that they have not seen before in the training data (Wei et al., 2023). However, our study shows that GPT-3.5 was unable to correctly identify the use of physics concepts in students' responses to physics problem-solving tasks without extensive prompt engineering. The used conventional ML model and the baseline classifier performed significantly better. Given our context, our results suggest that conventional ML models can be better adapted to a gold standard especially when expert-coded data is available. Of course, these models are then only suitable for a narrow range of applications and cannot handle the breadth of tasks that LLMs do. However, these smaller models offer further advantages in terms of transparency, processing speed, and energy consumption. Therefore, specialized ML models could be a more efficient and precise alternative in certain contexts. Especially in contexts in which there are not many different tasks to manage. It is important to remember that bigger is not necessarily better and it depends on research context whether conventional ML or LLMs are the optimal solution.

Related to designing teaching and learning environments for physics problem solving, our findings suggest that conventional ML models can be a valuable resource for automated classification. This is an important prerequisite for feedback systems that potentially enhance students' physics problem-solving abilities. Especially constructed response item formats such as the one evaluated in this study are an important means to enable students' to outline their cognitive processes related to physics problem solving. Automated analysis of these responses could enable online tutoring systems to report back the extent to which students' correctly represented a physics problem. In physics, robust application of physics concepts for solving problems is crucial for expertise development (Polverini and Gregorcic, 2024). Identifying concept use with ML and LLMs as presented in this study might pave the path toward developing tutoring systems that enable students to build this expertise.

## Data Availability

Publicly available datasets were analyzed in this study. This data can be found here: https://osf.io/d68ch/.

## Footnotes

^1^We present the word lists in German and English. Note that not all words can be translated as single words and that other language-specific details might be lost in the translation:force = (“gewichtskraft,” “gewichts-,” “zentripetalkraft,” “zentripetal-,” “zentrifugalkraft,” “zentrifugal-,” “flugkraft,” “flug-,” “erdanziehungskraft,” “erdanziehungs-,” “anziehungskraft,” “anziehungs-,” “gravitationskraft,” “gravitations-,” “fliehkraft,” “flieh-,” “radialkraft,” “radial-,” “schwerkraft,” “schwer-,” “zentralkraft,” “zentral-,” “gravitation,” “mindestgeschwindigkeit,” “kräftegleichgewicht,” and “kreisbewegung”); force (translated) = (“weight force,” “weight,” “centripetal force,” “centripetal,” “centrifugal force,” “centrifugal,” “flying force,” “flying,” “gravitational force,” “gravitational pull,” “gravitational,” “attractive force,” “attractive,” “radial force,” “radial,” “gravity,” “central force,” “central,” “gravitation,” “minimum speed,” “balance of forces,” and “circular motion”);energy = (“energie,” “kinetisch,” “potentiell,” “potenziell,” “energieerhaltungssatz,” “startpunkt,” “energieerhaltung,” “lageenergie,” “bewegungsenergie,” “höhendifferenz,” “gesamtenergie,” “höhepunkt,” “rotationsenergie,” “runterfallen,” and “herunterfallen”);energy (translated) = (“energy,” “kinetic,” “potential,” “energy conservation law,” “starting point,” “energy conservation,” “potential energy,” “kinetic energy,” “height difference,” “total energy,” “maximum height,” “rotational energy,” “fall down,” and “fall off”).

^2^As we singled out in Section 3.2, arguing about centripetal force in the inertial frame-of-reference would be more coherent, however, since almost no student did so, we used the balance of forces in the co-moving frame-of-reference here.
